# VentRa: distinguishing frontotemporal dementia from psychiatric disorders

**DOI:** 10.1093/braincomms/fcae069

**Published:** 2024-02-27

**Authors:** Ana L Manera, Mahsa Dadar, Simon Ducharme, D Louis Collins

**Affiliations:** McConnell Brain Imaging Centre, Montreal Neurological Institute, McGill University, Montreal, QC H3A 2B4, Canada; McConnell Brain Imaging Centre, Montreal Neurological Institute, McGill University, Montreal, QC H3A 2B4, Canada; Department of Psychiatry, Douglas Mental Health University Health Centre, McGill University, Montreal, QC H4H 1R3, Canada; McConnell Brain Imaging Centre, Montreal Neurological Institute, McGill University, Montreal, QC H3A 2B4, Canada; Department of Psychiatry, Douglas Mental Health University Health Centre, McGill University, Montreal, QC H4H 1R3, Canada; McConnell Brain Imaging Centre, Montreal Neurological Institute, McGill University, Montreal, QC H3A 2B4, Canada

**Keywords:** frontotemporal dementia, dementia, brain imaging, lateral ventricles, classification, psychiatric disorder

## Abstract

The volume of the lateral ventricles is a reliable and sensitive indicator of brain atrophy and disease progression in behavioural variant frontotemporal dementia. In this study, we validate our previously developed automated tool using ventricular features (known as VentRa) for the classification of behavioural variant frontotemporal dementia versus a mixed cohort of neurodegenerative, vascular and psychiatric disorders from a clinically representative independent dataset. Lateral ventricles were segmented for 1110 subjects—14 behavioural variant frontotemporal dementia, 30 other frontotemporal dementia, 70 Lewy body disease, 898 Alzheimer’s disease, 62 vascular brain injury and 36 primary psychiatric disorder from the publicly accessible National Alzheimer’s Coordinating Center dataset to assess the performance of VentRa. Using ventricular features to discriminate behavioural variant frontotemporal dementia subjects from primary psychiatric disorders, VentRa achieved an accuracy rate of 84%, a sensitivity rate of 71% and a specificity rate of 89%. VentRa was able to identify behavioural variant frontotemporal dementia from a mixed age–matched cohort (i.e. other frontotemporal dementia, Lewy body disease, Alzheimer’s disease, vascular brain injury and primary psychiatric disorders) and to correctly classify other disorders as ‘not compatible with behavioral variant frontotemporal dementia’ with a specificity rate of 83%. The specificity rates against each of the other individual cohorts were 80% for other frontotemporal dementia, 83% for Lewy body disease, 83% for Alzheimer’s disease, 84% for vascular brain injury and 89% for primary psychiatric disorders. VentRa is a robust and generalizable tool with potential usefulness for improving the diagnostic certainty of behavioural variant frontotemporal dementia, particularly for the differential diagnosis with primary psychiatric disorders.

## Teaser

Manera *et al*. report that their tool, VentRa, achieved an 84% accuracy rate in distinguishing behavioural variant frontotemporal dementia from psychiatric disorders. With 71% sensitivity and 89% specificity rates against primary psychiatric disorders, VentRa exhibits promise for behavioural variant frontotemporal dementia diagnosis, aiding in differentiating from a diverse cohort, including neurodegenerative and vascular disorders.

## Graphical Abstract

**Graphical Abstract:**
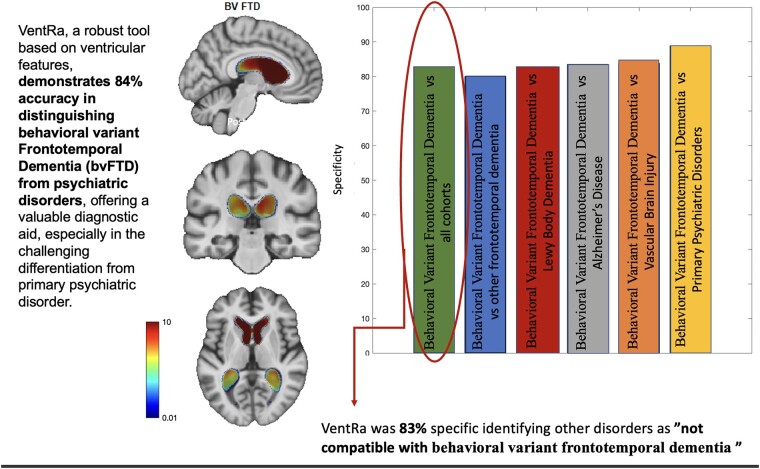

## Introduction

A confirmed behavioural variant frontotemporal dementia (bvFTD) diagnosis^1-3^ is often difficult to achieve in the clinic and it heavily relies on brain imaging. This is explained in part by the high heterogeneity in heritability, pathology and the absence of a unique molecular biomarker. In addition, the symptomatic overlap with other neurodegenerative disorders, but more importantly with non-degenerative primary psychiatric disorders (PPDs)—including major depressive disorder, bipolar disorder, schizophrenia, obsessive–compulsive disorder, autism spectrum disorders and even personality disorders^4^—means that PPDs often constitute the main differential diagnosis of bvFTD.^5^ It has been reported that ∼50% of patients with bvFTD receive a prior psychiatric diagnosis (most frequently major depression), and the average diagnostic delay is up to 5–6 years from symptom onset.^4,6^ It has also been shown that patients with PPDs can be wrongly diagnosed with bvFTD, particularly in community settings, preventing patients from having access to evidence-based psychiatric treatments.^7^

In prior research utilizing deformation-based morphometry, we demonstrated the robustness of ventricles as a metric for distinguishing bvFTD from cognitively normal controls.^8^ This study employed an atlas-based analysis to offer regional insights into cerebral changes, revealing that the lateral ventricles display the most notable volumetric difference at baseline between bvFTD and cognitively normal controls. Additionally, they exhibit the most significant progression of change in the 1-year follow-up among all structures studied.

In more recent work, we assessed the differences in ventricular features among bvFTD, normal controls and other dementia cohorts and developed VentRa, the first tool to use ventricular features specifically for the differential diagnosis of bvFTD. These features included total ventricular volume, left–right frontal ventricle volume ratio, left–right temporal ventricle volume ratio and anterior–posterior ratio (APR). Using VentRa, we achieved a 10-fold cross-validation accuracy rate of 80% (76% sensitivity and 83% specificity rates) in differentiating bvFTD from all other cohorts that included normal ageing, mild cognitive impairment, Alzheimer’s dementia and other frontotemporal dementia (FTD) variants. Interestingly, using only the APR ventricular feature yielded a 76% accuracy rate, underlying its importance in differentiating between groups.^9^ The lateral ventricles can be segmented reliably through manual efforts or with commonly available tools.^10,11^ They offer a reliable measure of overall brain atrophy across diverse regions, positioning VentRa’s ventricle-based features as a promising and practical tool for diagnosing bvFTD in both research and clinical settings.

In the present study, we aimed to validate VentRa on an independent cohort obtained from the National Alzheimer’s Coordinating Center (NACC) dataset to differentiate bvFTD subjects from other FTD, Lewy body disease (LBD), Alzheimer’s dementia, vascular brain injury (VBI) and PPDs. While the key differential diagnosis is between bvFTD variants and PPDs, we assessed the performance of VentRa in data from patients with other neurodegenerative dementias (other FTD subtypes, Alzheimer’s dementia, LBD and VBI) to verify that VentRa can detect atrophy patterns specific to bvFTD and not just any neurodegenerative disease–related pattern of atrophy that might also occur in other dementias. The publicly available NACC dataset was selected as the target dataset for independent validation of VentRa since it contains a large number of multi-centre and multi-scanner datasets with T_1_-weighted (T_1_-W) images acquired with different acquisition protocols, field of strength, resolution and field of view, allowing us to validate the generalizability of VentRa in realistic clinical settings.

## Materials and methods

### Participants

**Figure 1:**
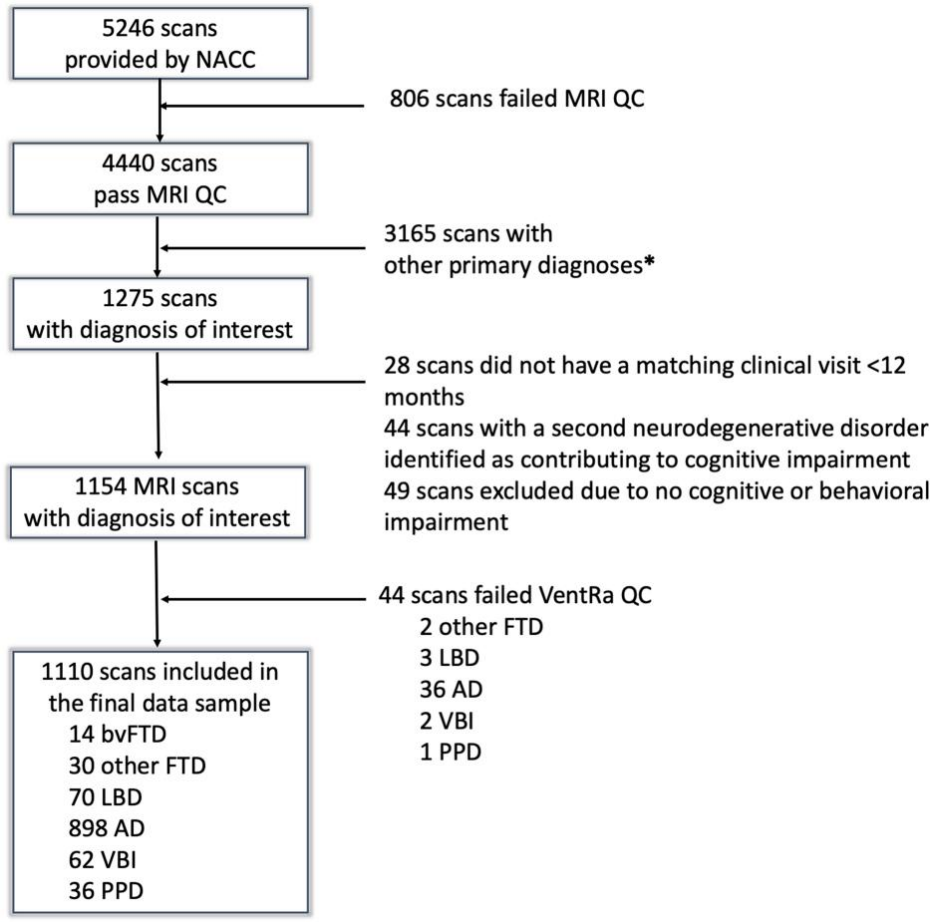
**A flowchart showing the subject selection procedure.** *A total of 144 scans were excluded due to missing diagnosis, 1931 due to no cognitive impairment and 1090 due to primary diagnoses other than the groups of interest (i.e. progressive supranuclear palsy, corticobasal degeneration, prion disease, traumatic brain injury, normal-pressure hydrocephalus, central nervous system neoplasm and other neurologic, genetic or infectious condition). The number of patients for each of these primary diagnoses that was excluded was very small and precluded in comparison with bvFTD.

The data sample used for this study was obtained from the NACC’s Uniform Data Set. The NACC developed a database of standardized clinical research data obtained from past and present National Institute on Aging–funded Alzheimer’s Disease Research Centers across the USA. NACC data collection has been previously described elsewhere.^12^ The participants included in the database had a range of cognitive statuses: normal cognition, mild cognitive impairment and demented due to various clinical diagnoses. A total of 5246 scans were obtained from the NACC from the September 2019 data freeze after a data request for all participants with any dementia syndrome (i.e. bvFTD, Alzheimer’s dementia, VBI, LBD and other), mild cognitive impairment and cognitively normal subjects who had at least one T_1_-W scan ±6 months from a clinical visit (NACC variables MRIT1 = 1 and NACCMRDY between −180 and 180). Figure 1 shows a flowchart of the detailed subject selection procedure. The analysis reported in this study used data from 1110 subjects from 6 diagnostic groups of interest—bvFTD (*n* = 14), other FTD (*n* = 30), LBD (*n* = 70), Alzheimer’s dementia (*n* = 898), VBI (*n* = 62) and PPDs (*n* = 36)—that passed quality control (QC) and had an available scan matching a clinical visit <12 months apart. The PPD group was composed of subjects with different psychiatric disorders: depression (*n* = 28), bipolar disorder (*n* = 2), anxiety disorder (*n* = 2) and other psychiatric diseases (*n* = 5). Of note, subjects who did not have any cognitive and/or behavioural impairment (*n* = 51) were excluded from this sample according to the following NACC variables: (i) NACCCOGF = 0 (no impairment in cognition), (ii) COGMODE = 0 (no impairment in cognition), (iii) NACCBEHF = 0 (no behavioural symptoms, for the bvFTD cohort) and (iv) COMPORT = 0.0 (no impairment in behaviour, comportment and personality, for the bvFTD cohort). As outlined in the documentation provided by the NACC database, the decision-making process for a presumptive aetiologic diagnosis of the cognitive disorder incorporated clinical diagnostic criteria, brain imaging and biomarkers.

### MRI acquisition parameters

Of all the participants selected for the final data sample, there were 529 3.0 T and 449 1.5 T scans acquired by GE (*n* = 575), SIEMENS (*n* = 189) or Philips (*n* = 81) scanners (no data on the field strength were available for 132 scans). Supplementary Table 2 shows the number of subjects in each cohort per strength field scanner. The scans were acquired in sagittal, coronal and axial planes from T_1_-W turbo field echo, fast spin echo, fast spoiled gradient-echo or magnetization-prepared rapid gradient echo sequences. Other acquisition parameters were as follows: echo time range: 0.0013–0.02; repetition time range: 0.006–3.00; the number of slices in *x* range: 124–512; the number of slices in *y* range: 54–512; the number of slices in *z* range: 29–512; slice thickness range: 0.8–5; voxel size *x* range: 0.4–1.2; voxel size *y* range: 0.4–3; and voxel size *z* range: 0.5–7.

### Statistical analysis

All statistical analyses were executed using MATLAB (version R2021b). Categorical variables’ differences among cohorts were evaluated using a *χ*^2^ test, while continuous variables underwent one-way ANOVA or Kruskal–Wallis variance analysis, depending on variable distribution. *Post hoc* two-sample Student’s *t*-tests were performed for examining clinical and ventricular distinctions. Results are presented as mean ± standard deviation and median (interquartile range) as appropriate. The *P*-values for volumetric analyses were adjusted for multiple comparisons using the Bonferroni method; any reported adjusted *P*-value < 0.05 is considered statistically significant.

### VentRa tool

VentRa takes a comma-separated (.csv) file with paths for raw T_1_-W images, along with subjects’ age and sex as input. It produces pre-processed images, ventricle segmentations, QC files and a .csv file containing diagnosis (from the classifier trained on bvFTD versus mixed group data) and extracted ventricle features. These features include total ventricle volume, volumes in each lobe and hemisphere, APR and left/right ratios. VentRa employs a support vector machine classifier with ventricular features (APR, total ventricular volume, left–right frontal ventricle volume ratio and left–right temporal ventricle volume ratio) along with age and sex. This classifier, originally trained on a separate dataset,^9^ remains unchanged and is tested on an independent dataset in this study.

To assess the robustness of the performance of VentRa with respect to the field strength of the input T_1_-W images, we ran VentRa on data from 100 individuals from the Alzheimer’s Disease Neuroimaging Initiative dataset that had T_1_-W scans available from both 1.5 and 3 T scanners that were acquired <10 days apart. VentRa was independently run on these scans, and we assessed the consistency of the obtained ventricle segmentations using the Dice similarity index as well as volumetric correlations. The 1.5- and 3-T segmentations had excellent agreement, with an average Dice similarity index of 0.963 ± 0.014 and a correlation of 0.998. Supplementary Figure 1 shows an example of the 1.5- and 3-T T_1_-W images and their corresponding segmentations in the stereotaxic space.

### Ventricular feature estimation and analyses

VentRa includes a previously validated patch-based label fusion technique to segment the lateral ventricles.^11^ The method uses expert manual segmentations as priors and estimates the label of each test subject voxel by comparing its surrounding 3D voxel patch against all the patches from the training library and performing a weighted label fusion using the intensity-based distances between the patch under study and the patches in the training subjects. All resulting segmentations were visually assessed by an experienced rater blind to the clinical diagnosis, and the incomplete/inaccurate segmentations (*n* = 44 or 3.96%) were excluded (Fig. 1). While some of these could be salvaged by manual corrections, we decided to evaluate only those with an objective automatic segmentation. Using the Hammers brain atlas delineating the frontal, parietal, temporal and occipital lobes separately in the left and right hemispheres,^13,14^ the lateral ventricular volumes were calculated per each brain lobe and hemisphere. Using a coronal coordinate *y* = −12 mm in the stereotaxic space (i.e. registered to the template), the ventricles were divided into anterior and posterior portions to obtain the ventricular APR.^9^ All volumes were normalized for intra-cranial volume, and these ratios were log-transformed to achieve normal distribution.

## Results

### Demographics

Table 1 shows demographic and cognitive testing performances for all the cohorts. The bvFTD subjects were younger than LBD, Alzheimer’s dementia and VBI subjects, and the age difference was not significant among bvFTD, other FTD and PPDs. There were no significant differences in years of cognitive impairment onset between the groups. There were no significant differences between bvFTD and all the other cohorts in the mini-mental status examination (MMSE; *P* > 0.05). bvFTD subjects were slightly more severely impaired than those with Alzheimer’s dementia according to clinical dementia rating-sum of boxes (CDR-SB; *P* = 0.05) and significantly more impaired than those with other FTD (*P* = 0.01), VBI (*P* < 0.001) and PPDs (*P* < 0.001). However, on average, they remained mild cases (CDR 1.0) of bvFTD and at a stage relevant for differential diagnosis. Other cognitive scores can be found in Supplementary Table 1.

**Table 1:** Demographic and clinical characteristics of all the cohorts

	BvFTD (*N* = 16)	Other FTD (*N* = 30)	LBD (*N* = 70)	Alzheimer’s dementia (*N* = 898)	VBI (*N* = 62)	PPD (*N* = 36)	*P*-value
Age, years	62 ± 7	66 ± 10	74 ± 7	75 ± 9	79 ± 8	69 ± 10	**<0.001**
Sex, male, *n* (%)	9 (56%)	13 (43%)	59 (84%)	441 (49%)	39 (63%)	19 (53%)	**<0.001**
Years from cognitive impairment onset	2.8 (2.3–6.3)	3 (2.3–5.5)	4.6 (3.3–6.8)	4.3 (2.8–6.4)	3.9 (2.1–6)		0.08
MMSE (*n* = 860)	24 (21–28)	25 (21–28)	26 (20–28)	24 (20–27)	27 (24–28)	28 (26–29)	**<0.001**
CDR Global score (*n* = 1110)	1 (0.5–1)	0.5 (0.5–1)	0.5 (0.5–1)	0.5 (0.5–1)	0.5 (0.5–1)	0.5 (0.5–0.5)	**<0.001**
CDR-SB (*n* = 1110)	5.25 (4.5–9)	2.75 (1–4.5)	3 (2–7)	3.5 (1.5–5)	1.5 (1–2.5)	1 (0.5–2)	**<0.001**

Values express mean ± SD/median (interquartile range). *P*-value level of significance: 0.05. The bold values represent significant differences with the bvFTD cohort.

### Classification task with VentRa

#### bvFTD versus PPDs

**Figure 2:**
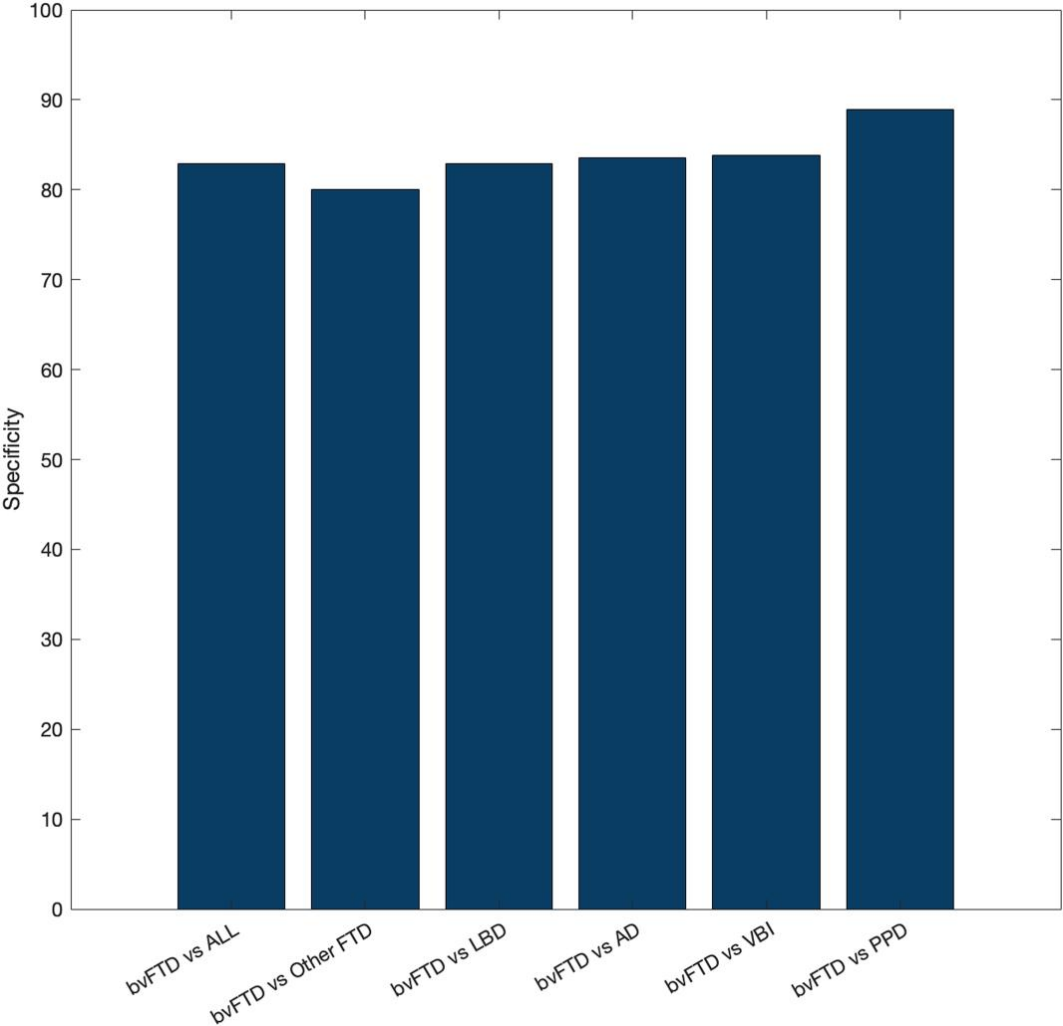
**Specificity for the different classification tasks.** ALL, mixed cohort.

VentRa achieved an accuracy rate of 84%, a sensitivity rate of 71% and a specificity rate of 89% in discriminating bvFTD subjects from PPDs based on ventricular features (Fig. 2). Positive and negative likelihood ratios were 6.43 and 0.31, respectively.

**Figure 3:**
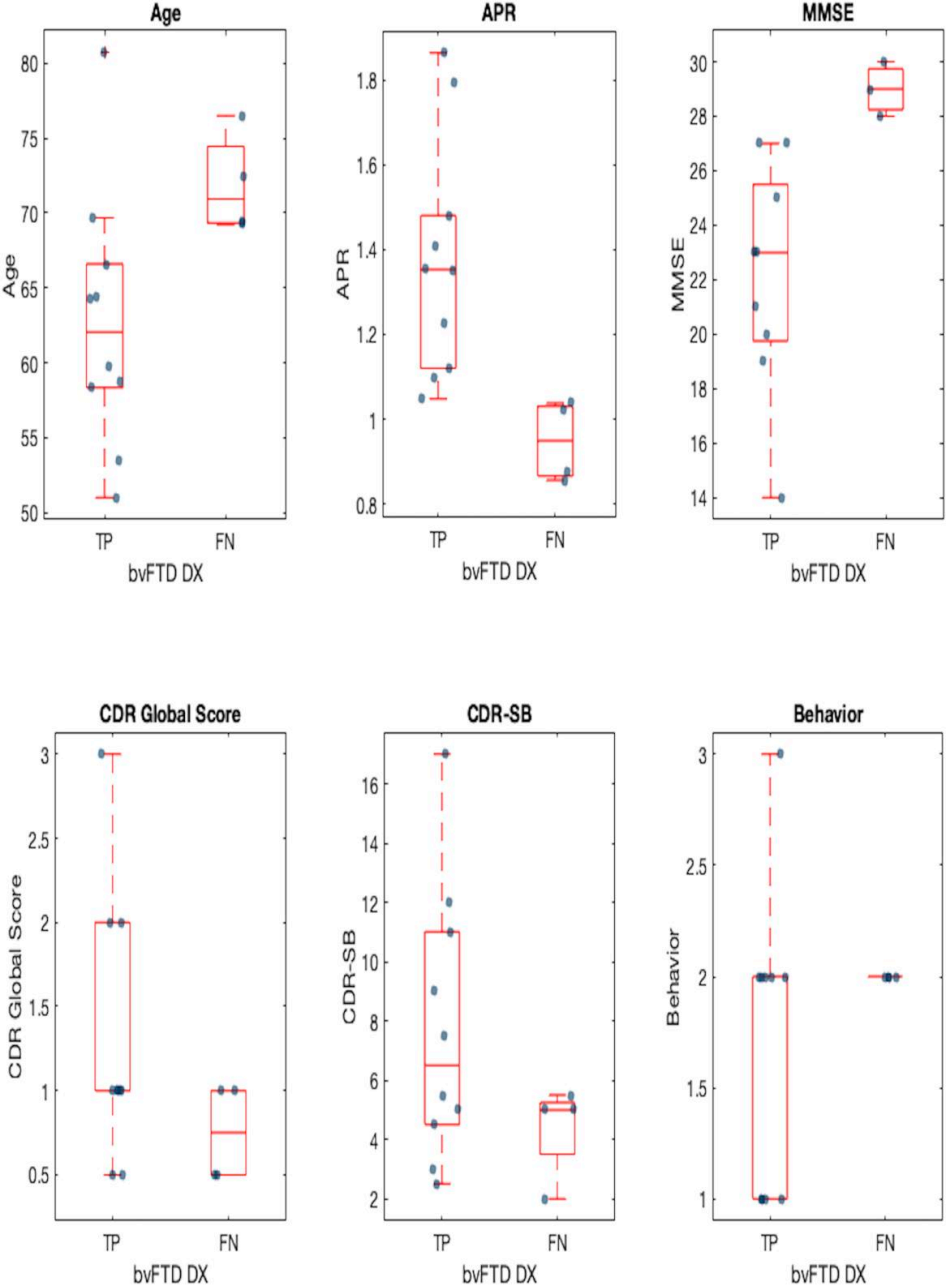
**Boxplots showing the differences between bvFTD subjects correctly classified with VentRa (TPs) and those classified as not compatible with bvFTD (FNs).** AGE_TP_ 63 ± 8 years versus AGE_FN_ 72 ± 3 years (*P* = 0.06). APR_TP_ was significantly larger than APR_FN_ (*P* = 0.01). The FN also performed significantly better than the TPs on MMSE (*P* = 0.02). Median CDR Global_TP_ = 1 and median CDR Global_FN_ = 0.75 (*P* = 0.2). Median CDR-SB_TP_ = 6.5 and median CDR-SB_FN_ = 5 (*P* = 0.2). No significant differences were found in the severity of behaviour, comportment and personality impairment (*P* = 0.4). Behaviour refers to the NACC variable ‘COMPORT’ (behaviour, comportment and personality impairment), where 0 = no impairment, 0.5 = questionable impairment, 1 = mild impairment, 1.5 = moderate impairment and 2 = severe impairment. DX, diagnosis.

The classification using VentRa resulted in a 29% rate of false negatives (FNs). As shown in Fig. 3, these subjects were older than the bvFTD subjects correctly classified (72 ± 3 versus 63 ± 8 years, respectively, *P* = 0.06), and they had a significantly smaller ventricular APR (*P* = 0.01). The FN also performed significantly better than the true positives (TPs) on MMSE (*P* = 0.02). Yet, the disease duration was not significantly different from TPs (*P* = 0.2) nor was the severity according to the CDR Global score (median CDR Global_TP_ = 1 and median CDR Global_FN_ = 0.75, *P* = 0.2) and CDR-SB (median CDR-SB_TP_ = 6.5 and median CDR-SB_FN_ = 5, *P* = 0.2). Finally, no significant differences were found in the severity of behaviour, comportment and personality impairment (*P* = 0.4) according to the NACC variable ‘COMPORT’, where 0 = no impairment, 0.5 = questionable impairment, 1 = mild impairment, 1.5 = moderate impairment and 2 = severe impairment.

**Figure 4:**
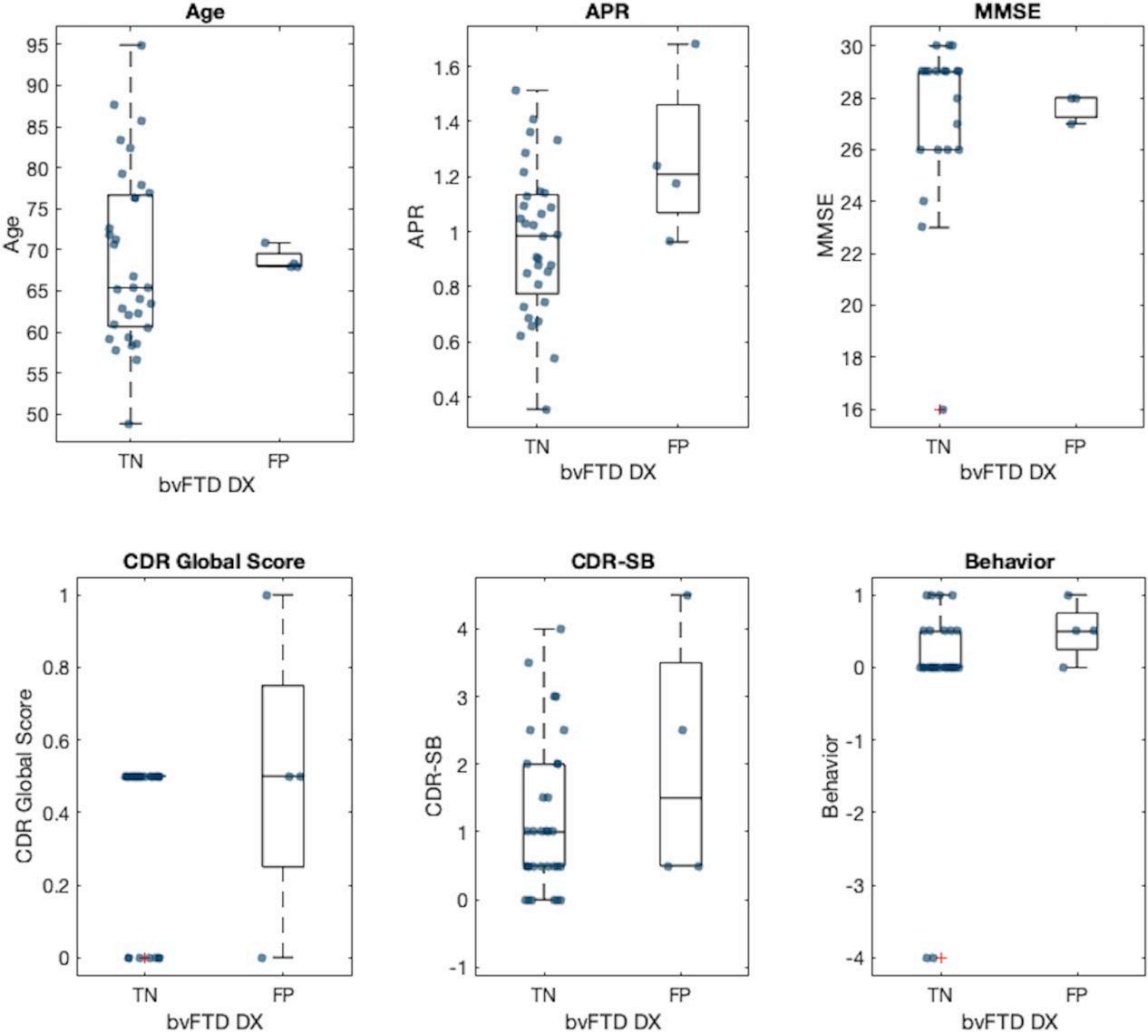
**Boxplots showing the differences between PPD subjects incorrectly classified as bvFTD with VentRa (FPs) and those classified as not compatible with bvFTD (TNs).** Behaviour refers to the NACC variable ‘COMPORT’ (behaviour, comportment and personality impairment), where 0 = no impairment, 0.5 = questionable impairment, 1 = mild impairment, 1.5 = moderate impairment and 2 = severe impairment. DX, diagnosis; TN, true negative.

On the other hand, the false-positive (FP) rate was 11%. When comparing the FP against the PPD subjects correctly diagnosed as not compatible with bvFTD (Fig. 4), no significant differences were found in age (*P* = 0.9), MMSE (*P* = 0.8), CDR (*P* = 0.4), CDR-SB (*P* = 0.2) or the severity of behavioural impairment (‘COMPORT’, *P* = 0.33). The FP showed a statistically significant larger APR compared with those subjects who were correctly classified (1.26 ± 0.3 and 0.97 ± 0.3, respectively, *P* = 0.04).

#### bvFTD versus other neurodegenerative cohorts

Using ventricular features to identify bvFTD from a mixed age–matched cohort (‘ALL’ = other FTD, LBD, Alzheimer’s dementia, VBI and PPDs), VentRa was able to correctly classify other disorders as ‘not compatible with bvFTD’ with a specificity rate of 83%. The specificity rates against each of the other individual cohorts were 80% for other FTD, 83% for LBD, 83% for Alzheimer’s dementia and 84% for VBI (Fig. 2).

## Discussion

In the present study, we aimed to validate our previously developed automated tool VentRa^9^ for the estimation of ventricular features on T_1_-W MRI for the individual prediction of bvFTD within a mixed cohort of subjects from an independent dataset of clinically acquired MRIs. The main findings were as follows: (i) VentRa was able to accurately differentiate bvFTD from PPDs with high specificity and moderate sensitivity rates (84% accuracy, 71% sensitivity and 89% specificity); (ii) VentRa was able to identify bvFTD from a mixed cohort with a specificity rate of 83%; (iii) from each of the other individual cohorts (other FTD, LBD, Alzheimer’s dementia and VBI), VentRa correctly identified between 80 and 84% as not compatible with bvFTD; and (iv) our results showed good positive and negative likelihood ratios, proving its reliability to rule in and rule out bvFTD among the wider group of patients exhibiting behavioural changes such as apathy, disinhibition and loss of empathy.^15^

While current clinical diagnostic criteria for the different neurodegenerative disorders in combination with several available pathologic biomarkers (i.e. Alzheimer’s dementia biomarkers in blood/cerebrospinal fluid and positron emission tomography) perform reasonably well, the main clinical challenge in the differential diagnosis of bvFTD is still represented by PPDs. Clinical criteria for bvFTD are less helpful to distinguish it from PPDs.^5,16^ Bedside cognitive screening lacks the ability to effectively distinguish between bvFTD and PPDs. Neuropsychological assessment frequently focuses more on the executive dysfunction characteristic of bvFTD, although it is highly non-specific for FTD versus PPDs.^16^ Despite suggestions of its efficacy, a dedicated assessment of social cognition is often not included in standard neuropsychological assessment batteries.^16,17^ Furthermore, several social cognitive instruments that have been developed for research purposes are yet to have normative data available.^18^

Neuroimaging is already crucial for bvFTD diagnosis; frontal and/or anterior temporal atrophy or hypometabolism increases certainty from possible to probable bvFTD in current criteria.^1^ Yet, MRIs may not be obviously abnormal in early stages or in certain variants of bvFTD, such as the C9orf72 phenotype.^16,19^ In the Late-Onset Frontal Lobe Syndrome study, the standard visual review of structural MRI was found to be useful for the differential diagnosis of bvFTD versus PPDs; however, it had only a 70% sensitivity rate.^20,21^ It is hypothesized that volumetric MRI could improve this sensitivity rate; however, there is insufficient evidence currently to formally recommend volumetric analytic techniques in clinical populations with behavioural changes.^18^ Furthermore, while some machine learning algorithms using MRI volumetry perform well against controls and Alzheimer’s dementia cohorts, less is known about the performance against PPDs. Zhutovsky *et al*.^22^ performed a classification of bvFTD versus PPDs with different data usage approaches, including clinical, MRI voxel-wise, MRI region of interest, clinical + MRI region of interest and clinical + MRI voxel-wise techniques. In their study, a support vector machine was trained on a sample of 73 patients (18 bvFTD, 28 neurological and 27 psychiatric). Zhutovsky *et al*.^22^ reported cross-validation accuracy rates of 82.1% (76.7% sensitivity, 87.5 specificity) and 78.3% (75.9% sensitivity, 80.8% specificity) using only MRI voxel-wise and MRI region of interest approaches, respectively. Cross-validation can overestimate performance as the same cohort is used to select features and optimize the method’s hyperparameters. In contrast, our validation using *independent* data shows that our method generalizes well, achieving 84% accuracy, 71% sensitivity and 89% specificity in discriminating bvFTD subjects from PPDs with VentRa. This rate of accuracy with a higher specificity relative to sensitivity is in line with the accuracy rate from an expert neuroradiological visual review reported in the Late-Onset Frontal Lobe Syndrome cohort.^20^ Although the performance of VentRa was not superior to others, the fact that it was completely automated and performed as well as academic centre experts could lead to an improvement in the quality of clinical practice, particularly in community areas. Further, VentRa has the potential to improve over time if more training data are included.

Still, our results showed an FN rate of 29%. This was likely because these FN bvFTD subjects were older than the TP subjects and outside the age range of bvFTD subjects from the Frontotemporal Lobar Degeneration Neuroimaging Initiative that was originally used to train VentRa (age ranges: bvFTD_NIFD_ 61 ± 6 years, TP_NACC_ 63 ± 8 years and FN_NACC_ 72 ± 3 years). Since these subjects were outside the operating range of the classifier, they were more likely to be misclassified, thus underlying the importance of having training data that well represent the population of interest. It is highly probable that adding extended ages to the training dataset will likely improve the results. Not only were the FN subjects older but also they had better cognition (MMSE, CDR Global and CDR-SB), and their ventricular APR was smaller; we might therefore argue that there could be a mismatch between the clinical population included in the NACC (including some older and milder cases, possibly with more ambiguous diagnoses) and the Frontotemporal Lobar Degeneration Neuroimaging Initiative data (more advanced and clear-cut cases) used to train VentRa. We also need to consider the fact that in older-age FTD, there is a higher prevalence of mixed pathologic diagnoses,^23^ and therefore, a classification algorithm is unlikely to perform as well in this population.

Our study has two main strengths. First, VentRa has been trained and cross-validated on a sample from two different multi-centre databases (Frontotemporal Lobar Degeneration Neuroimaging Initiative^24^ and Alzheimer’s Disease Neuroimaging Initiative),^25^ and its performance was tested here on a held-out database that also included multi-centre and multi-scanner data from different scanner models of both 1.5 and 3 T field strengths (NACC). In addition, the failure rate of VentRa QC was very low (4%), particularly considering the variability of MRI acquisition parameters (i.e. slice thickness range: 0.8–5, voxel size *x* range: 0.4–1.2, voxel size *y* range: 0.4–3 and voxel size *z* range: 0.5–7). This further reinforces the generalizability (i.e. external validity) of our results and ensures their applicability in a clinical scenario with different scanners and with different magnetic field strengths. This raises confidence that VentRa would work reliably in clinical settings. Secondly, our tool is based on standard structural T_1_-W MRI and uses a combination of easily measurable features of the lateral ventricles, which can be reliably segmented using a variety of publicly available tools such as FreeSurfer in addition to the patch-based method used in VentRa.^10,11^ Further, all the image processing tools used in this study have been established and validated for use in multi-centre and multi-scanner datasets^8,14,26-29^ and have been designed to minimize such centre-related and scanner-related differences. The ventricular features are much less likely to be impacted by such differences, particularly for the APR, where any such discrepancies would be minimized and perhaps cancelled in the ratio.

Our study has notable limitations, particularly related to the small sample size of bvFTD subjects. The number of bvFTD subjects with available and good-quality scans was relatively limited. In subsequent research, it is crucial to evaluate the robustness and generalizability of our results in larger and more diverse bvFTD cohorts and to compare the clinical utility of VentRa with expert radiological assessment.^2,30^ Additionally, it is essential to investigate how VentRa performs in a more heterogeneous PPD cohort and also its ability to predict the future course in patients with clinically ambiguous FTD presentations. (i.e. predict who has a neurodegenerative underlying pathology versus who has a PPD). The current small sample size underscores the need for caution in interpreting and applying our results. Since we have made both the image processing pipeline and the classification model publicly available with VentRa (http://nist.mni.mcgill.ca/VentRa/), this can be easily achieved by others who might have access to such datasets. Finally, replicating these results in a cohort with pathologic/genetic confirmation, ensuring a definite bvFTD diagnosis, is crucial for establishing the tool’s reliability and applicability.

## Conclusion

These results prove the robustness and generalizability of VentRa, indicating the potential usefulness of this automated MRI-based tool for improving the diagnostic certainty of bvFTD. If validated in a prospective larger sample, VentRa has the potential to improve diagnostic accuracy, particularly against populations with behavioural and/or psychiatric symptoms, enabling reduced diagnostic and intervention time, particularly in settings with less access to specialized neuroradiology.

## Supplementary Material

fcae069_Supplementary_Data

## Data Availability

Data elements from the National Alzheimer’s Coordinating Center (NACC) dataset are publicly available. The ventricle feature estimation and classification tool developed in this project (VentRa) is publicly available at: http://nist.mni.mcgill.ca/?p=2498. Other raw data from this study are available upon reasonable request to the authors.
