# Supplementary material for: VentRa: distinguishing frontotemporal dementia from psychiatric disorders
Source: Brain Commun. 2024 Feb 27;6(2):fcae069. doi: 10.1093/braincomms/fcae069 (PMC10953623; doi:10.1093/braincomms/fcae069)
Supplement: fcae069_Supplementary_Data [file fcae069_supplementary_data.docx]

**Supplementary materials**

|  | bvFTD  (N=16) | Other FTD (N=30) | LBD  (N=70) | AD  (N=898) | VBI  (N=62) | PPD  (N=36) | *p* value |
| --- | --- | --- | --- | --- | --- | --- | --- |
| Age, y | 62±7 | 66±10 | **74±7** | **75±9** | **79±8** | 69±10 | <0.001 |
| Sex, male (%) | 9 (56%) | 13 (43%) | 59 (84%) | 441 (49%) | 39 (63%) | 19 (53%) | <0.001 |
| Years from cognitive impairment onset, y | 2.8[ 2.3-6.3] | 3 [2.3-5.5] | 4.6 [3.3-6.8] | 4.3 [2.8-6.4] | 3.9 [2.1-6] | - | 0.08 |
| MMSE  (n= 860) | 24 [21-28] | 25 [21-28] | 26 [20-28] | 24 [20-27] | 27 [24-28] | 28 [26-29] | <0.001 |
| CDR Global score (n=1110) | 1 [0.5-1] | **0.5 [0.5-1]** | 0.5 [0.5-1] | 0.5 [0.5-1] | **0.5 [0.5-1]** | **0.5 [0.5-0.5]** | <0.001 |
| CDR-SB  (n=1110) | 5.25 [4.5-9] | **2.75 [1-4.5]** | 3 [2-7] | **3.5 [1.5-5]** | **1.5 [1-2.5]** | **1[0.5-2]** | <0.001 |
| TRAIL-A time, s  (n=1041) | 37 [23-64] | 45 [33-63] | 56 [40-85] | 44 [32-66] | 55 [41-75] | 38 [31-54] | <0.001 |
| TRAIL-B time, s  (n=902) | 76 [66-90] | 111 [81-179] | **171 [111-300]** | **149 [99 -266]** | **204 [116-292]** | 103 [64-170] | <0.001 |
| Digits forward correct trials  (n=852) | 6 [5 -9] | 6 [4-6] | 8 [7-9] | 7 [6-9] | 7 [6-8] | 8 [6-10] | 0.01 |
| Digits backwards (n=850) | 5 [4-7] | 5 [4-6] | 4 [3-5] | 5 [4-6] | 5 [4-6] | 6 [4-7] | 0.04 |
| WAIS  (n=767) | 43 [29-46] | 38 [24-48] | 26 [21-35] | 32 [22-42] | 29 [19-34] | 35 [27-45] | 0.002 |
| BOSTON  (n=443) | 20 [17-23] | 22 [11-29] | 24 [21-26] | 22 [16-25] | 23 [18-26] | 26 [21-27] | 0.02 |
| Semantic Fluency – Animals  (n=1081] | 11 [4-13] | 11 [6-18] | 12 [7-16] | 12 [9-16] | 13 [11-16] | **16 [13-19]** | <0.001 |
| Semantic Fluency – Vegetables  (n=1078] | 6 [3-8] | 8 [4-12] | 7 [1-10] | 8 [5-11] | 10 [7-11] | **12 [9-16]** | <0.001 |

**Supplementary table 1. Demographic and clinical characteristics for all the cohorts.** Values express Mean± SD / Median [interquartile range]. P value level of significance: 0.05. Abbreviations: bvFTD: behavioral variant frontotemporal dementia; FTD: Frontotemporal dementia; LBD: Lewy Body Disease; AD: Alzheimer’s dementia; VBI: Vascular Brain Injury; PPD: Primary Psychiatric Disorder; MMSE: Mini-Mental Status Examination; CDR: Clinical Dementia Rating; CDR-SB: Clinical Dementia Rating-Sum of Boxes. The bold values represent significant differences with the bvFTD cohort.


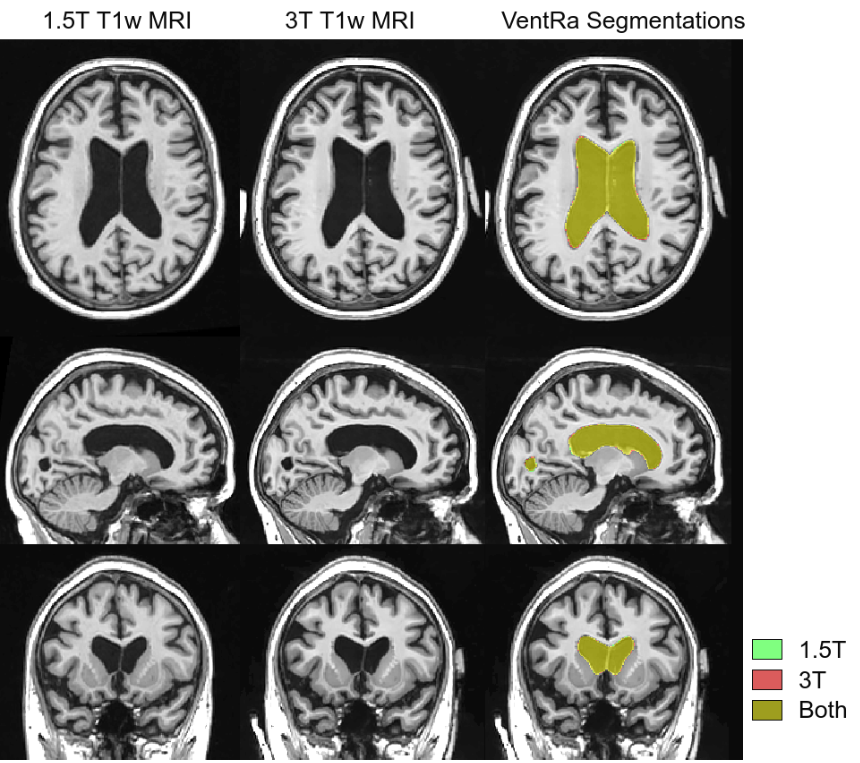


**Supplementary Figure 1:** Corresponding segmentations of 1.5T and 3T T1w images in the stereotaxic space.

|  | bvFTD  (N=16) | Other FTD (N=30) | LBD  (N=70) | AD  (N=898) | VBI  (N=62) | PPD  (N=36) |
| --- | --- | --- | --- | --- | --- | --- |
| 1.5T ,n(%) | 3 (18%) | 6 (30%) | 45 (64%) | 368 (41%) | 24 (39%) | 14 (39%) |
| 3T ,n(%) | 9 (56%) | 24 (70%) | 25 (36%) | 530 (59%) | 38 (61%) | 22 (61%) |
| , Other/Missing/Unknown, n(%) | 4 (26%) |  |  |  |  | - |

**Supplementary Table 2:** Number of subjects in each diagnostic category per different strength fields (1.5T, 3T, or other/missing/unknown scanner information)
